# Characteristics and outcomes of patients with do-not-resuscitate and physician orders for life-sustaining treatment in a medical intensive care unit: a retrospective cohort study

**DOI:** 10.1186/s12904-024-01375-w

**Published:** 2024-02-15

**Authors:** Song-I Lee, Ye-Rin Ju, Da Hyun Kang, Jeong Eun Lee

**Affiliations:** Division of Pulmonary and Critical Care Medicine, Department of Internal Medicine, Chungnam National University School of Medicine, Chungnam National University Hospital, 282 Munhwa-ro, Jung-gu, Daejeon, 35015 Republic of Korea

**Keywords:** Do not resuscitate, Medical treatment, Life-sustaining treatment, Risk factors

## Abstract

**Background:**

In the intensive care unit (ICU), we may encounter patients who have completed a Do-Not-Resuscitate (DNR) or a Physician Orders to Stop Life-Sustaining Treatment (POLST) document. However, the characteristics of ICU patients who choose DNR/POLST are not well understood.

**Methods:**

We retrospectively analyzed the electronic medical records of 577 patients admitted to a medical ICU from October 2019 to November 2020, focusing on the characteristics of patients according to whether they completed DNR/POLST documents. Patients were categorized into DNR/POLST group and no DNR/POLST group according to whether they completed DNR/POLST documents, and logistic regression analysis was used to evaluate factors influencing DNR/POLST document completion.

**Results:**

A total of 577 patients were admitted to the ICU. Of these, 211 patients (36.6%) had DNR or POLST records. DNR and/or POLST were completed prior to ICU admission in 48 (22.7%) patients. The DNR/POLST group was older (72.9 ± 13.5 vs. 67.6 ± 13.8 years, *p* < 0.001) and had higher Acute Physiology and Chronic Health Evaluation (APACHE) II score (26.1 ± 9.2 vs. 20.3 ± 7.7, *p* < 0.001) and clinical frailty scale (5.1 ± 1.4 vs. 4.4 ± 1.4, *p* < 0.001) than the other groups. Solid tumors, hematologic malignancies, and chronic lung disease were the most common comorbidities in the DNR/POLST groups. The DNR/POLST group had higher ICU and in-hospital mortality and more invasive treatments (arterial line, central line, renal replacement therapy, invasive mechanical ventilation) than the other groups. Body mass index, APAHCE II score, hematologic malignancy, DNR/POLST were factors associated with in-hospital mortality.

**Conclusions:**

Among ICU patients, 36.6% had DNR or POLST orders and received more invasive treatments. This is contrary to the common belief that DNR/POLST patients would receive less invasive treatment and underscores the need to better understand and include end-of-life care as an important ongoing aspect of patient care, along with communication with patients and families.

**Supplementary Information:**

The online version contains supplementary material available at 10.1186/s12904-024-01375-w.

## Introduction

The concept of “quality of death” is increasingly recognized as being as important as quality of life. In response, South Korea’s National Assembly passed the Life-Sustaining Treatment Decision Act in 2016, which has been gradually implemented since 2017. This law provides a framework for patients to make informed decisions about their end-of-life care, emphasizing respect for patient autonomy. It enables patients to plan their medical care in advance through an ‘Advance Directive for Life-Sustaining Treatment’. If patients are unable to communicate their wishes, the law allows family members to make decisions on their behalf. These directives are registered with the National Life-Sustaining Treatment Management Agency and discussed with healthcare professionals, ensuring that patients’ preferences are respected in critical situations. This change in the law has led to a growing interest in and understanding of life-sustaining treatment among critically ill patients.

Cancer patients are generally more interested in and prepared for end-of-life care than patients with other diseases. Reflecting this, a study in South Korea examined the impact of life-sustaining treatment decision act on the end-of-life care of cancer patients [1]. The study by Won et al. found that 26.4% of these patients complied with documentation of limiting life-sustaining treatment, but this documentation did not significantly alter decisions to withhold or withdraw treatment [1]. This suggests that while cancer patients are proactive in preparing for end-of-life care, their documented preferences may not always directly influence treatment decisions. Park et al. found that factors such as gender, age, living in non-metropolitan areas, and comorbidities also play a significant role in shaping end-of-life decisions [2], suggesting a complex interplay of demographic and health-related factors in end-of-life care.

In a study conducted in South Korean intensive care units (ICU) following the implementation of the Life-sustaining Treatment decision Act, haemato-oncology was the most common department to withhold or withdraw life-sustaining treatment. The greater the involvement of intensivists in end-of-life decisions, the higher the rate of treatment withdrawal and transfer from the ICU to the ward, suggesting that they play an important role in guiding families and avoiding unnecessary treatment [3]. Im et al. also demonstrated that the frequency of cardiopulmonary resuscitation (CPR) did not change before and after the implementation of life-sustaining treatment decision act [4]. These findings suggest that balancing legal changes with clinical judgement in treatment decisions can be challenging. For example, Do-Not-Resuscitate (DNR) orders in septic patients are associated with poor prognosis [5], but the timing of DNR orders does not appear to affect prognosis [5, 6], suggesting the need to think about the timing and decision-making process in critical care.

These findings not only reflect the state of end-of-life care in South Korea, but also provide valuable lessons for global healthcare systems facing similar ethical dilemmas. The South Korean context of a rapidly ageing society with an increasing proportion of elderly patients adds another layer to this discussion [7]. Older age is known to be associated with increased ICU mortality [8–10] and may influence DNR and life-sustaining treatment decisions [11–13]. However, there may be gaps in knowledge regarding the baseline characteristics and clinical outcomes of medical ICU patients according to whether they have completed a DNR or POLST. This study therefore aims to explore these gaps and provide a better understanding of how they affect critical care decisions and outcomes.

## Material and method

**Fig. 1:**
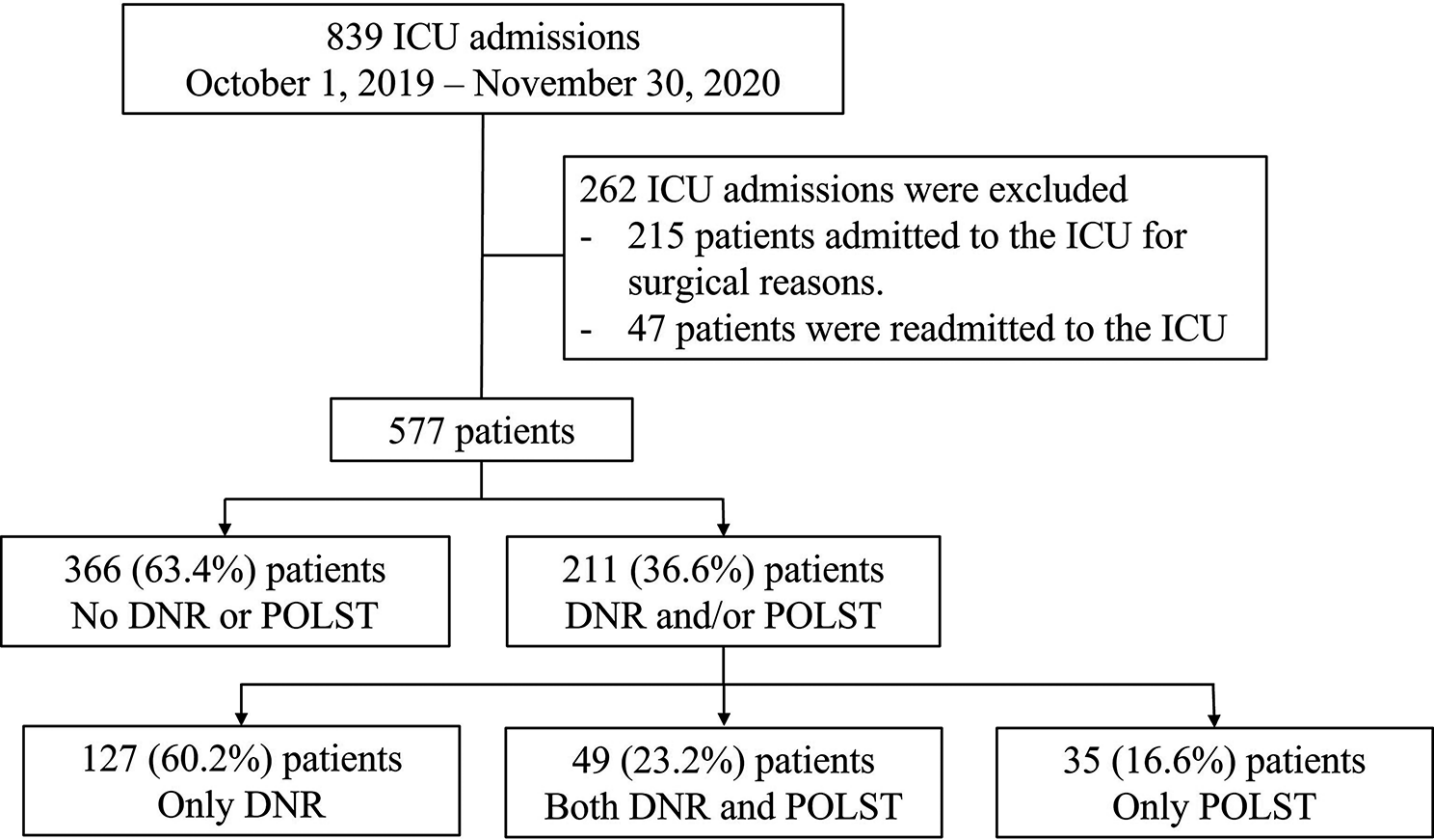
Flow chart of enrolled patients. ICU, intensive care unit; DNR, do not resuscitate; POLST, physician orders for life-sustaining treatment

All data for our study were obtained from electronic medical records (EMR, C&U Care 2.0). Between October 1, 2019, and November 30, 2020, 839 patients were admitted to the medical ICU. We included 577 of these patients in our study, specifically excluding 215 who were admitted for surgical reasons due to the different characteristics and needs of surgical patients compared to medical ICU patients. In addition, 47 patients who were readmitted to the ICU were excluded to avoid duplication of data and because readmitted patients typically have a worse prognosis, which could bias our results (Fig. 1). DNR orders are used to avoid unnecessary procedures, such as intubation and CPR, for patients whose treatment has been deemed futile or who are in a situation where CPR is expected. Physician Orders to Stop Life-Sustaining Treatment (POLST) forms are prepared to avoid certain procedures on patients, including CPR, hemodialysis, chemotherapy, tracheal intubation, and the use of mechanical ventilation. In this study, all patients who completed POLST forms were patients who declined all of the above.

The Institutional Review Board (IRB) of Chungnam national university hospital (IRB No: CNUH 2021-07-041) approved this study. The informed consent requirement was waived due to retrospective study design.

### Collection of data

Patient age, sex, underlying disease, and the patient baseline data were collected from the EMR. The Acute Physiology and Chronic Health Evaluation (APACHE) II score on ICU admission and initial clinical frailty scale before ICU admission were collected. Documented data were collected on DNR and POLST, timing of documentation, and interventions performed during ICU admission (arterial line, central line, vasopressors, high-flow nasal cannula, invasive mechanical ventilation, extracorporeal membrane oxygenation, continuous renal replacement therapy [CRRT], and tracheostomy). We also collected the duration of ICU stay and mortality, in-hospital duration of stay, and in-hospital mortality for the evaluation of patient prognoses.

We divided the patients in our analysis into two groups: those who had documented DNR and/or POLST forms (DNR/POLST group) and those who had no DNR or POLST documentation at all (no DNR/POLST group). We conducted additional analyses specifically on the DNR/POLST group. Within this group, we defined patients with only DNR documentation as the DNR group, and those with POLST documentation or both POLST and DNR documentation as the POLST group.

### Statistical analysis

Data was analyzed according to the type of variable. Continuous variables were expressed as mean ± standard deviation, and Student’s t-test or Mann-Whitney U test was used. Categorical variables were expressed as percentages and Pearson’s chi-squared test or Fisher’s exact test was used. Univariate and multivariate logistic regression analysis was performed to evaluate the factors associated with disease severity. Adjusted odds ratios (OR) and 95% confidence intervals (CI) were used to represent variables independently associated with disease severity. P-value < 0.05 was considered statistically significant. We performed all statistical analyzes using SPSS software (version 22.0; IBM Corporation, Somers, NY, USA).

## Results

### Clinical characteristics of patients

A total of 577 patients admitted to the ICU were enrolled during the study period (October 1, 2019, to November 30, 2020), except 215 patients admitted to the ICU for surgical reasons and 47 patients readmitted to the ICU. A total of 366 patients (no DNR/POLST group, 63.4%) did not write a document on DNR or POLST, while 211 patients (DNR/POLST group, 36.6%) wrote a document on DNR and/or POLST (Fig. 1).

Table 1 presents the patient’s baseline characteristics. The patients in the DNR/POLST group were older (72.9 ± 13.5 vs. 67.6 ± 13.8 years, *p* < 0.001), and the APACHE II score (26.1 ± 9.2 vs. 20.3 ± 7.7, *p* < 0.001) and clinical frailty scale (5.1 ± 1.4 vs. 4.4 ± 1.4, *p* < 0.001) were higher than those in the no DNR/POLST group. In the underlying disease, solid tumor (16.1% vs. 8.2%, *p* = 0.004), hematologic malignancy (3.3% vs. 0.8%, *p* = 0.027), and chronic lung disease (30.8% vs. 18.9%, *p* = 0.001) were more frequent in the DNR/POLST group than in the no DNR/POLST group. Laboratory findings revealed that hemoglobin and albumin levels were lower and total bilirubin and c-reactive protein (CRP) levels were greater in the DNR/POLST group than in the no DNR/POLST group.

**Table 1:** Baseline characteristics of enrolled patients

Characteristics	All patients	DNR/POLST	No DNR/POLST	P-value
Patients (n)	577	211	366	
Age, yr	69.5 ± 13.9	72.9 ± 13.5	67.6 ± 13.8	< 0.001
Male	348 (60.3)	119 (56.4)	229 (62.6)	0.145
Body mass index, kg/m^2^	22.8 ± 3.9	22.6 ± 4.2	22.9 ± 3.7	0.370
APACHE II score	22.6 ± 8.8	26.1 ± 9.2	20.3 ± 7.7	< 0.001
Clinical frailty scale	4.6 ± 1.4	5.1 ± 1.4	4.4 ± 1.4	< 0.001
**Underlying disease**				
Hypertension	343 (59.4)	121 (57.3)	222 (60.7)	0.435
Diabetes Mellitus	238 (41.2)	88 (41.7)	150 (41.0)	0.865
Solid tumor	64 (11.1)	34 (16.1)	30 (8.2)	0.004
Hematologic malignancy	10 (1.7)	7 (3.3)	3 (0.8)	0.027
Chronic heart disease	194 (33.6)	69 (32.7)	125 (34.2)	0.722
Chronic lung disease	134 (23.2)	65 (30.8)	69 (18.9)	0.001
Chronic liver disease	61 (10.6)	23 (10.9)	38 (10.4)	0.845
Cerebrovascular accident	95 (16.5)	42 (19.9)	53 (14.5)	0.091
Chronic kidney disease	55 (9.5)	22 (10.4)	33 (9.0)	0.579
**Laboratory findings**				
White blood cell, ×10^3^/uL	10.7 (7.3–15.8)	11.3 (7.3–17.1)	10.4 (7.3–15.4)	0.130
Hemomglobin, g/dL	10.9 (9.4–12.9)	10.2 (9.1–11.8)	11.3 (9.7–13.4)	< 0.001
Platelet, ×10^3^/uL	179 (120–243)	166 (97–235)	184 (128–245)	0.071
Total bilirubin, mg/dL	0.7 (0.5–1.2)	0.8 (0.5–1.4)	0.7 (0.5–1.1)	0.003
Albumin, g/dL	2.9 (2.5–3.5)	2.7 (2.3–3.0)	3.1 (2.6–3.6)	< 0.001
AST, U/L	33 (21–82)	36 (23–114)	31 (20–72)	0.308
ALT, U/L	23 (14–51)	24 (13–55)	23 (14–50)	0.457
Creatinine, mg/dL	1.14 (0.69–2.17)	1.25 (0.72–2.18)	1.08 (0.68–2.19)	0.152
CRP, ng/mL	5.2 (0.8–14.8)	7.7 (1.2–17.4)	3.5 (0.6–12.4)	0.003

Data are presented as mean ± standard deviation or median and interquartile range or number (%), unless otherwise indicated

DNR, do-not-resuscitate; POLST, physician orders for life sustaining treatment; APACHE II, Acute physiology and chronic health evaluation; AST, aspartate aminotransferase; ALT, alanine aminotransferase; CRP, C-reactive protein

### Treatment and prognosis of patients

Table 2 shows patient outcomes and interventions performed in the ICU. The DNR/POLST group received more arterial lines (94.8% vs. 79.5%, *p* < 0.001), central lines (81.5% vs. 60.9%, *p* < 0.001), CRRT (29.4% vs. 15.6%, *p* < 0.001), and invasive mechanical ventilation (73.0% vs. 48.4%, *p* < 0.001) in the ICU than did the no DNR/POLST group.

**Table 2:** Outcomes and interventions of the patients

Characteristics	All patients	DNR/POLST	No DNR/POLST	P-value
**Interventions in the ICU**				
Arterial line	491 (85.1)	200 (94.8)	291 (79.5)	< 0.001
Central line	395 (68.5)	172 (81.5)	223 (60.9)	< 0.001
Vasopressors	323 (56.0)	118 (55.9)	205 (56.0)	0.984
Continuous renal replacement therapy	119 (20.6)	62 (29.4)	57 (15.6)	< 0.001
HFNC	538 (93.2)	193 (91.5)	345 (94.3)	0.198
Invasive mechanical ventilation	331 (57.4)	154 (73.0)	177 (48.4)	< 0.001
ECMO	12 (2.1)	2 (0.9)	10 (2.7)	0.148
Tracheostomy	57 (9.9)	27 (12.8)	30 (8.2)	0.075
**Outcomes**				
ICU mortality	119 (20.6)	107 (50.7)	12 (3.3)	< 0.001
ICU stay, days	6.0 (3.0–12.0)	8.0 (3.0–16.0)	5.0 (3.0–9.0)	< 0.001
In-hospital mortality	157 (27.2)	134 (63.5)	25 (6.8)	< 0.001
Hospital stay, days	17.0 (9.0–36.0)	15.0 (7.0–39.0)	17.0 (9.0–35.0)	0.828
Duration of mechanical ventilation (*n* = 331)	4.0 (2.0–11.0)	7.0 (2.0–15.0)	3.0 (1.0–8.5)	0.001

Data are presented as mean ± standard deviation or median and interquartile range or number (%), unless otherwise indicated

DNR, do-not-resuscitate; POLST, physician orders for life sustaining treatment; ICU, intensive Care Unit; HFNC, high flow nasal cannula; ECMO, extracorporeal membrane oxygenation

Additionally, the DNR/POLST group had higher ICU mortality (50.7% vs. 3.3%, *p* < 0.001) and higher in-hospital mortality (63.5% vs. 6.8%, *p* < 0.001); a longer ICU length of stay (8.0 [3.0–16.0] vs. 5.0 [3.0–9.0] days, *p* < 0.001); and longer duration of mechanical ventilation (7.0 [2.0–15.0] vs. 3.0 [1.0–8.5] days, *p* = 0.001) than the no DNR/POLST group.

### Identification of factors associated with in-hospital mortality

Table 3 shows the results of the multivariate statistical analysis of the factors related to in-hospital mortality. After adjustment of the confounders, predictors of in-hospital mortality included body mass index (BMI, ORs, 1.054; 95% CI, 1.017–1.093; *p* = 0.004), APACHE II score (ORs, 1.025; 95% CI, 1.005–1.046; *p* = 0.015), hematologic malignancy (ORs, 2.686; 95% CI, 1.239–5.824; *p* = 0.012), and patients who completed DNR/POLST (ORs, 10.353; 95% CI, 6.261–17.119; *p* < 0.001).

**Table 3:** Univariate and multivariate Cox regression analysis addressing the risk factors for in-hospital mortality

	Univariate analysis			Multivariate analysis		
	OR	95% CI	P-value	OR	95% CI	P-value
Age	1.005	0.993–1.017	0.381			
Male	0.942	0.685–1.294	0.711			
BMI	1.064	1.024–1.106	0.002	1.054	1.017–1.093	0.004
APACHE II score	1.056	1.039–1.074	< 0.001	1.025	1.005–1.046	0.015
Clinical frailty scale	1.249	1.122–1.390	< 0.001	1.089	0.971–1.221	0.147
**Underlying disease**						
Solid tumor	1.786	1.205–2.648	0.004	1.018	0.670–1.546	0.934
Hematologic malignancy	3.324	1.553–7.111	0.002	2.686	1.239–5.824	0.012
Chronic lung disease	1.391	0.990–1.953	0.057	1.024	0.707–1.483	0.899
**Laboratory findings**						
White blood cell, ×10^3^/uL	1.003	0.989–1.018	0.671			
Hemomglobin	0.972	0.912–1.035	0.374			
Platelet, ×10^3^/uL	0.999	0.997–1.001	0.218			
Total bilirubin, mg/dL	1.057	1.010–1.107	0.016	0.991	0.934–1.050	0.756
Albumin, g/dL	0.558	0.427–0.729	< 0.001	0.909	0.662–1.249	0.556
CRP, ng/mL	1.009	0.993–1.026	0.281			
DNR and/or POLST	9.477	6.179–14.535	< 0.001	10.353	6.261–17.119	< 0.001
Invasive mechanical ventilation	2.536	1.701–3.781	< 0.001	1.506	0.956–2.372	0.078
Vasopressor	0.913	0.668–1.248	0.568			
CRRT	1.978	1.432–2.732	< 0.001	1.245	0.879–1.764	0.218

BMI, body mass index; APACHE II, Acute physiology and chronic health evaluation; CRP, c-reactive protein; DNR, do-not-resuscitate; POLST, physician orders for life sustaining treatment; CRRT, continuous renal replacement therapy

### Characteristics of patients who wrote the DNR or POLST

Additional Table 1 shows the baseline patient characteristics. The DNR group showed higher APACHE II score than that in the POLST group. Moreover, hypertension was slightly less common in the DNR group. However, there was no statistical difference in terms of age, male sex, BMI, or clinical frailty scale between the two groups. Furthermore, no statistically significant differences were observed between the two groups in terms of laboratory findings.

Additional Table 2 shows the ICU interventions and outcomes in the patient group that wrote the document of DNR/POLST. There were more cases in which DNR or POLST documents were written before ICU admission in the POLST group than in the DNR group (32.9% vs. 12.5%, *p* < 0.001). No statistically significant differences were observed between the two groups in terms of ICU intervention and outcomes. The duration from document completion to in-hospital death was longer in the POLST group (3.0 [1.0–16.0] vs. 2.0 [0.0–15.0], *p* = 0.027) than that in the DNR group.

The factors associated with in-hospital mortality in the DNR/POLST group are shown in Additional Table 3. The independent predictors of in-hospital mortality included BMI (OR, 1.067; 95% CI, 1.027–1.108; *p* = 0.001) and hematologic malignancy (OR, 2.382; 95% CI, 1.027–5.526; *p* = 0.043). DNR/POLST documentation completed prior to ICU admission was not an independent factor of in-hospital mortality.

## Discussion

This study compared the characteristics, interventions, and prognoses of patients admitted to a medical ICU according to whether the DNR/POLST was written. Among patients admitted to the ICU, the DNR/POLST group was older, had a higher score of APACHE II, and was frailer. The DNR/POLST group had more underlying diseases, and laboratory findings included anemia, hyperbilirubinemia, hypoalbuminemia, and higher CRP. We predicted that the DNR/POLST group would receive fewer interventions, based on the assumption that these directives generally favour less aggressive medical care. However, contrary to our expectations, this group received more interventions and had a higher mortality rate. This discrepancy suggests a complex relationship between patients’ medical directives and the medical care they actually received in a critical situation.

The DNR/POLST group was older, more severely ill, and frailer. In addition, among the underlying diseases, the DNR/POLST group had more solid tumors, hematologic malignancies, and chronic lung diseases. Similar trends have been observed in other studies. In Chang et al.’s study of septic medical ICU patients, the DNR group was older, Charlson comorbidity index scores and APACHE II score were higher, and malignancy was more common than that in the without-DNR group [5]. A study by Huang et al. comprising patients with severe sepsis and septic shock also found that the DNR group was older, had higher sequential organ failure assessment score and APACHE II score, and had higher prevalence of diabetes mellitus and hypertension than the non-DNR group [14]. A study by Serrano-Eanelli et al. found that the group that completed life-sustaining treatment act document was older than the group that did not [15]. A study by Devanand et al. of patients admitted to an intensive care unit after an out-of-hospital cardiac arrest found that patients older than 65 years and those with higher Charlson comorbidity index and APACHE II scores were more likely to have a decision to withdraw life-sustaining therapy [16]. Taken together, these studies consistently show that DNR/POLST patients tend to be older, more severely ill, frail, and have a higher prevalence of comorbidities.

Initially, based on the general understanding of DNR/POLST guidelines, which often suggests limiting aggressive treatments [17, 18], we predicted that the DNR/POLST group in our study would undergo fewer medical interventions and potentially have higher mortality rates. Contrary to this, our findings revealed more interventions such as arterial lines, central lines, continuous renal replacement therapy, and invasive mechanical ventilation, along with higher ICU and in-hospital mortality, and extended ICU stays in the DNR/POLST group.

Comparatively, Vranas et al. [19] reported more hemodialysis and blood transfusions in the POLST group in emergency departments, although intubation/mechanical ventilation and cardiopulmonary resuscitation rates were similar between groups with and without POLST. Similarly, in a study by Lee et al. [20], the likelihood of intensive care unit admission, invasive mechanical ventilation, use of vasopressors, and cardiopulmonary resuscitation varied by the type of treatment restriction in the POLST. However, other studies showed no significant correlation between DNR/POLST and interventions like intubation and hemodialysis [5, 21]. In this study, the high incidence of intensive treatments observed in patients with DNR or POLST orders may be due to a specific practice in Korean ICUs. Here, rather than stopping all aggressive treatments, DNR or POLST is often used to prevent the administration of CPR when active treatment is ongoing, especially when the patient’s death appears imminent, and CPR would likely be ineffective. This shows that despite DNR/POLST guidelines, approaches to end-of-life care can vary greatly depending on local medical practices and patient conditions. The variability in ICU and in-hospital mortality and length of stay seen in several studies [5, 19, 20, 22] further emphasizes the complexity of these decisions and their consequences in different settings.

In this study, we identified body mass index, APACHE II score, hematologic malignancy, and DNR/POLST as significant factors associated with in-hospital mortality in critically ill patients. These findings are consistent with previous studies [23–28] that highlighted various prognostic factors, including age, medical reason for hospitalization, gender, need for a ventilator, and specific levels of ferritin and vitamin B12. In addition, scoring systems such as the Simple Acute Physiology Score and APACHE II have also been recognized as key indicators of patient outcomes. Other studies have also observed the impact of treatment restrictions due to early implementation of DNR and POLST directives [5, 12, 19]. When considering the combination of these factors, it becomes clear that patients in the DNR/POLST group are more likely to face a more challenging prognosis. These insights underscore the importance of considering multiple clinical and ethical factors when managing and treating ICU patients, including comorbidities, severity of illness as indicated by various scores, and the presence of DNR/POLST directives. This comprehensive approach is critical to making informed decisions and optimizing patient care in the critical care setting.

This study further aimed to assess whether there were any notable differences between patients in the DNR group and the POLST group. The study found no significant differences in age, underlying medical conditions, or initial test results between the two groups. However, the frequency of DNR/POLST documentation prior to ICU admission was higher in the POLST group, and the time from documentation to death was significantly shorter in the DNR group. The only difference observed in terms of medical interventions was the use of invasive mechanical ventilation. There were no differences in ICU and in-hospital mortality rates and ICU and hospital length of stay. The increase in pre-ICU documentation in the POLST group is likely due to increased interest in end-of-life care following the enactment of the Health Care for Life Act, which led to an increase in the number of individuals completing such documents [1, 29, 30], influencing the prevalence of POLST completion prior to ICU admission. As for DNRs, they are often completed to avoid unnecessary CPR in the face of imminent death, so it is likely that the interval from completion to death is shorter in the group that did not complete a POLST compared to the DNR group.

While the concept of a “good death” is not universally defined, there is widespread agreement that it should preserve human dignity, be pain-free, and occur in the presence of loved ones [31]. This understanding emphasizes the importance of healthcare providers working with patients to determine their preferred place of death and the scope of care they want, especially for patients nearing the end of life [32]. Even after the implementation of the life-sustaining-treatment act, there have been cases where patients who had written an advance directive were admitted to the intensive care unit and received a similar level of intervention as patients without an advance directive. In Korea, ICU hospitalization and invasive interventions often occur against the patient’s wishes, especially if the family is unaware of or does not agree with the patient’s wishes regarding end-of-life care [33]. Therefore, it is important for healthcare providers to discuss POLST with both patients and families prior to ICU admission to clarify the meaning and scope of treatment. These conversations are necessary to help make sure that a patient’s end-of-life care is consistent with their wishes and values, paving the way for what can be considered a “good death” [34].

There are several limitations to this study. First, as a retrospective, single-center study, we relied only on the electronic medical record, which has some inherent limitations in data collection. This approach may have resulted in missing data that were not recorded. In addition, although our hospital is a tertiary center that treats a varied patient population, a single-center study may not fully represent the full range of ICU patient groups commonly seen in multiple centers. Second, the sample size of our study was relatively small; however, after consultation with the Department of Statistics, we determined that the sample size was sufficient to maintain statistical significance and the integrity of our findings. Third, our study did not assess family members’ satisfaction with and involvement in DNR or POLST decisions. While these aspects may provide valuable insights into the decision-making process and its impact on patients’ and families’ experiences, they were not possible within the current limits of our study.

## Conclusions

In conclusion, the study found that 36.6% of patients admitted to a medical ICU had completed a DNR/POLST document. Notably, invasive interventions in the DNR/POLST group were similar to or higher than those in the group that did not complete a DNR/POLST, which is contrary to general expectations. This group also had higher ICU and in-hospital mortality rates, showing that DNR/POLST was significantly associated with prognosis. Interestingly, there was no significant difference in prognosis between the DNR and POLST groups. These findings highlight the complexity of end-of-life care decisions in the ICU and underscore the importance of timely and meaningful discussions about DNR/POLST among healthcare providers, patients, and families. These conversations are essential not only to tailor medical interventions to the patient’s preferences, but also to promote a dignified end-of-life experience. Therefore, we believe that consideration should be given to a more patient- and caregiver-centered approach to discussing and implementing DNR/POLST directives, which we believe is an important step in ensuring a “good death” that respects the patient’s wishes and dignity.

### Electronic supplementary material

Below is the link to the electronic supplementary material.

Supplementary Material 1. Additional Table 1. Baseline characteristics of patients depending on the document type. Additional Table 2. Outcomes and interventions of the patients depending on the document type. Additional Table 3. Univariate and multivariate Cox regression analysis addressing the risk factors for in-hospital mortality in the POLST and/or DNR group.

## Data Availability

The datasets used and/or analyzed during the current study are available from the corresponding author upon reasonable request.

## Abbreviations

APACHE: Acute Physiology and Chronic Health Evaluation
BMI: body mass index
CI: Confidence interval
CPR: Cardiopulmonary resuscitation
CRP: c-reactive protein
CRRT: continuous renal replacement therapy
DNR: do-not-resuscitate
ICU: intensive care unit
OR: Odds ratio
POLST: physician orders for life-sustaining treatment
