# Supplementary material for: Characteristics and outcomes of patients with do-not-resuscitate and physician orders for life-sustaining treatment in a medical intensive care unit: a retrospective cohort study
Source: BMC Palliat Care. 2024 Feb 15;23:42. doi: 10.1186/s12904-024-01375-w (PMC10868112; doi:10.1186/s12904-024-01375-w)
Supplement: Supplementary file 1 — Supplementary Material 1. Additional Table 1. Baseline characteristics of patients depending on the document type. Additional Table 2. Outcomes and interventions of the patients depending on the document type. Additional Table 3. Univariate and multivariate Cox regression analysis addressing the risk factors for in-hospital mortality in the POLST and/or DNR group. [file 12904_2024_1375_MOESM1_ESM.docx]

**Additional file**

Additional Table 1. Baseline characteristics of patients depending on the document type

| Characteristics | All patients | POLST | DNR | P-value |
| --- | --- | --- | --- | --- |
| Patients (n) | 211 | 84 | 127 |  |
| Age, yr | 72.9 ± 13.5 | 74.9 ± 10.8 | 71.6 ± 14.9 | 0.068 |
| Male | 119 (56.4) | 41 (48.8) | 78 (61.4) | 0.071 |
| Body mass index, kg/m^2^ | 22.6 ± 4.2 | 22.0 ± 3.6 | 23.0 ± 4.5 | 0.100 |
| APACHE II score | 26.1 ± 9.2 | 24.3 ± 8.0 | 27.3 ± 9.8 | 0.014 |
| Clinical frailty scale | 5.1 ± 1.4 | 5.0 ± 1.4 | 5.1 ± 1.4 | 0.643 |
| Underlying disease |  |  |  |  |
| Hypertension | 121 (57.3) | 55 (65.5) | 66 (52.0) | 0.052 |
| Diabetes Mellitus | 88 (41.7) | 38 (45.2) | 50 (39.4) | 0.397 |
| Solid tumor | 34 (16.1) | 15 (17.9) | 19 (15.0) | 0.575 |
| Hematologic malignancy | 7 (3.3) | 4 (4.8) | 3 (2.4) | 0.341 |
| Chronic heart disease | 69 (32.7) | 27 (32.1) | 42 (33.1) | 0.888 |
| Chronic lung disease | 65 (30.8) | 31 (36.9) | 34 (26.8) | 0.119 |
| Chronic liver disease | 23 (10.9) | 6 (7.1) | 17 (13.4) | 0.154 |
| Cerebrovascular accident | 42 (19.9) | 15 (17.9) | 27 (21.3) | 0.545 |
| Chronic kidney disease | 22 (10.4) | 5 (6.0) | 17 (13.4) | 0.084 |
| Laboratory findings |  |  |  |  |
| White blood cell, ×10^3^/uL | 11.3 (7.3 – 17.1) | 12.2 (7.9 – 17.2) | 11.1 (7.2 – 17.0) | 0.720 |
| Hemomglobin | 10.2 (9.1 – 11.8) | 10.1 (9.0 – 11.4) | 10.2 (9.1 – 12.5) | 0.706 |
| Platelet, ×10^3^/uL | 166 (97 – 235) | 167 (93 – 252) | 166 (101 – 229) | 0.676 |
| Total bilirubin, mg/dL | 0.8 (0.5 – 1.4) | 0.8 (0.5 – 1.2) | 0.8 (0.5 – 1.6) | 0.808 |
| Albumin, g/dL | 2.7 (2.3 – 3.0) | 2.7 (2.4 – 3.1) | 2.7 (2.3 – 3.0) | 0.416 |
| AST | 36 (23 – 114) | 34 (23 – 111) | 37 (23 – 120) | 0.797 |
| ALT | 24 (13 – 55) | 24 (12 – 57) | 24 (13 – 53) | 0.858 |
| Creatinine, mg/dL | 1.25 (0.72 – 2.18) | 1.04 (0.65 – 2.35) | 1.30 (0.79 – 2.14) | 0.525 |
| CRP, ng/mL | 7.7 (1.2 – 17.4) | 8.1 (1.0 – 18.7) | 7.7 (2.1 – 17.3) | 0.673 |

Data are presented as mean ± standard deviation or median and interquartile range or number (%), unless otherwise indicated.

POLST, physician orders for life-sustaining treatment; DNR, do-not-resuscitate; APACHE II, Acute physiology and chronic health evaluation; AST, aspartate aminotransferase; ALT, alanine aminotransferase; CRP, C-reactive protein

Additional Table 2. Outcomes and interventions of the patients depending on the document type

| Characteristics | All patients | POLST | DNR | P-value |
| --- | --- | --- | --- | --- |
| DNR or POLST completed before admitting the ICU | 48 (22.7) | 28 (32.9) | 22 (12.5) | <0.001 |
| Interventions in the ICU |  |  |  |  |
| Arterial line | 200 (94.8) | 81 (96.4) | 119 (93.7) | 0.383 |
| Central line | 172 (81.5) | 66 (78.6) | 106 (83.5) | 0.370 |
| Vasopressors | 118 (55.9) | 43 (51.2) | 75 (59.1) | 0.260 |
| Continuous renal replacement therapy | 62 (29.4) | 21 (25.0) | 41 (32.3) | 0.256 |
| HFNC | 193 (91.5) | 80 (95.2) | 113 (89.0) | 0.111 |
| Invasive mechanical ventilation | 154 (73.0) | 53 (63.1) | 101 (79.5) | 0.009 |
| ECMO | 2 (0.9) | 0 (0) | 2 (1.6) | 0.248 |
| Tracheostomy | 27 (12.8) | 7 (8.3) | 20 (15.7) | 0.115 |
| Outcomes |  |  |  |  |
| ICU mortality | 107 (50.7) | 44 (52.4) | 63 (49.6) | 0.693 |
| ICU stay, days | 8.0 (3.0 – 16.0) | 10.0 (4.0 – 18.8) | 7.0 (3.0 – 15.0) | 0.943 |
| In-hospital mortality | 134 (63.5) | 51 (60.7) | 83 (65.4) | 0.493 |
| Hospital stay, days | 15.0 (7.0 – 39.0) | 21.5 (13.3 – 49.3) | 11.0 (5.0 – 24.0) | 0.144 |
| Duration from the completion of the document to in-hospital death (n = 134) | 2.0 (0.0 – 15.0) | 3.0 (1.0 – 16.0) | 2.0 (0.0 – 15.0) | 0.027 |
| Duration of mechanical ventilation | 7.0 (2.0 – 15.0) | 9.5 (3.0 – 17.8) | 6.0 (2.0 – 11.0) | 0.608 |

Data are presented as median and interquartile range or number (%), unless otherwise indicated.

POLST, physician orders for life-sustaining treatment; DNR, do-not-resuscitate; ICU, intensive Care Unit; HFNC, high flow nasal cannula; ECMO, extracorporeal membrane oxygenation

Additional Table 3. Univariate and multivariate Cox regression analysis addressing the risk factors for in-hospital mortality in the POLST and/or DNR group

|  | Univariate analysis | | | Multivariate analysis | | |
| --- | --- | --- | --- | --- | --- | --- |
|  | OR | 95% CI | P-value | OR | 95% CI | P-value |
| Age | 0.990 | 0.978 – 1.002 | 0.106 |  |  |  |
| Male | 1.036 | 0.730 – 1.472 | 0.841 |  |  |  |
| BMI | 1.068 | 1.029 – 1.109 | 0.001 | 1.067 | 1.027 – 1.108 | 0.001 |
| APACHE II score | 1.023 | 1.004 – 1.042 | 0.018 | 1.008 | 0.988 – 1.028 | 0.435 |
| Clinical frailty scale | 1.011 | 0.896 – 1.140 | 0.858 |  |  |  |
| Underlying disease |  |  |  |  |  |  |
| Hypertension | 1.110 | 0.787 – 1.565 | 0.554 |  |  |  |
| Solid tumor | 1.283 | 0.853 – 1.931 | 0.232 |  |  |  |
| Hematologic malignancy | 2.617 | 1.137 – 6.024 | 0.024 | 2.382 | 1.027 – 5.526 | 0.043 |
| Chronic lung disease | 0.980 | 0.672 – 1.429 | 0.916 |  |  |  |
| Laboratory findings |  |  |  |  |  |  |
| White blood cell, ×10^3^/uL | 1.003 | 0.986 – 1.022 | 0.711 |  |  |  |
| Platelet, ×10^3^/uL | 1.001 | 0.999 – 1.002 | 0.523 |  |  |  |
| Total bilirubin, mg/dL | 1.006 | 0.953 – 1.062 | 0.815 |  |  |  |
| Albumin, g/dL | 0.875 | 0.627 – 1.222 | 0.433 |  |  |  |
| CRP, ng/mL | 0.993 | 0.975 – 1.013 | 0.503 |  |  |  |
| DNR or POLST completed before admitting the ICU | 0.590 | 0.362 – 0.962 | 0.034 | 0.646 | 0.385 – 1.085 | 0.099 |
| Invasive mechanical ventilation | 1.764 | 1.123 – 2.771 | 0.014 | 1.533 | 0.952 – 2.468 | 0.079 |
| Vasopressor | 1.301 | 0.922 – 1.835 | 0.134 |  |  |  |
| CRRT | 1.222 | 0.856 – 1.745 | 0.269 |  |  |  |

BMI, body mass index; APACHE II, Acute physiology and chronic health evaluation; CRP, c-reactive protein; DNR, do-not-resuscitate; POLST, physician orders for life sustaining treatment; CRRT, continuous renal replacement therapy
